# Reduced Serum Maresin-1 in Rheumatoid Arthritis and Fibromyalgia Compared with Healthy Controls: A Cross-Sectional Study

**DOI:** 10.5152/ArchRheumatol.2026.25249

**Published:** 2026-04-24

**Authors:** Türkan Tuncer, Nevsun Pıhtılı Taş, Muhammet Şahin Elbastı, Şeyma Demirelli

**Affiliations:** 1 Department of Physical Medicine and Rehabilitation, Elazığ Fethi Sekin City Hospital, Elazığ, Türkiye; 2 Elazığ Provincial Health Directorate Public Health Laboratory, Elazığ, Türkiye

**Keywords:** Fibromyalgia, inflammation, neuropathic pain, rheumatoid arthritis, serum Maresin-1

## Abstract

**Background/Aims:**

Maresin-1 (MaR-1), a specialized pro-resolving mediator, modulates inflammation and pain signaling. Serum MaR-1 was compared across rheumatoid arthritis (RA), fibromyalgia (FM), and healthy controls and examined its associations with disease activity and pain measures.

**Materials and Methods:**

In this cross-sectional study, patients with rheumatoid arthritis (RA) (n = 30), patients with fibromyalgia (FM) (n = 30), and healthy controls (n = 29) were enrolled. Clinical assessments included visual analog scale (VAS) scores for pain and fatigue, DAS28-CRP in the RA group, the PainDETECT questionnaire for neuropathic pain screening in RA patients, the Fibromyalgia Impact Questionnaire (FIQ) in the FM group, and the Beck Depression and Beck Anxiety Inventories (BDI and BAI) to assess emotional status. Serum MaR-1 was quantified by enzyme-linked immunosorbent assay. Group comparisons, correlations, and receiver-operating characteristic analyses were performed.

**Results:**

Controls had higher MaR-1 than both patient groups (*P* < .001). In RA, MaR-1 inversely correlated with VAS pain, DAS28; in FM, MaR-1 inversely correlated with VAS pain (*P* < .001). Discriminative ability in this cohort (RA): Area under the curve (AUC) 0.941 (95% CI 0.887-0.996), cut-off ≤1.07, sensitivity 86.7%, and specificity 93.1%. Discriminative ability in this cohort (FM): AUC 0.933 (95% CI 0.861-1.000), cut-off ≤1.19, sensitivity 90.0%, specificity 93.1%.

**Conclusion:**

Serum MaR-1 is reduced in RA and FM and tracks with pain severity and disease activity, supporting its potential as a biomarker of chronic inflammatory pain biology. Larger longitudinal studies are warranted to establish clinical utility.

**Main Points**

- Serum Maresin-1 (MaR-1) levels were reduced in patients with rheumatoid arthritis (RA) and fibromyalgia compared with healthy controls.
- Reduced MaR-1 levels were related to clinical disease activity in RA and symptom burden in fibromyalgia.
- Maresin-1 demonstrated group-level discriminative capacity in this study population.
- The results suggest a possible involvement of MaR-1 in inflammatory and nociplastic pain pathways.

## Introduction

Specialized pro-resolving mediators (SPMs) are bioactive lipid molecules synthesized from omega-3 or omega-6 fatty acids, which actively resolve inflammation and promote tissue homeostasis. The major groups of SPMs include resolvins, lipoxins, protectins, and maresins. Specialized pro-resolving mediators prevent the uncontrolled persistence of acute inflammation by inhibiting leukocyte migration and pro-inflammatory cytokine production, reprogramming macrophages toward a pro-resolving phenotype, and facilitating the clearance of apoptotic debris.^1^ They also accelerate tissue repair following injury, prevent excessive fibrosis, and limit permanent tissue damage. In adaptive immune cells, SPMs contribute to resolving inflammation and re-establishing immune homeostasis.^2^ Recent evidence indicates that SPMs exert neuroprotective effects by attenuating neuroinflammation in various neurodegenerative conditions.^1^

The primary precursor of Maresin-1 (MaR-1) is docosahexaenoic acid (DHA), an omega-3 fatty acid.^3^ Docosahexaenoic acid is oxidized by 12-lipoxygenase (12-LOX) in macrophages to form a hydroperoxide intermediate (14S-HpDHA), which undergoes further enzymatic conversion to MaR-1 via an epoxide intermediate.^3^ Maresin-1’s analgesic effects involve several mechanisms. After neural injury or inflammatory stimuli, glial cells—especially microglia and astrocytes—become activated and release IL-1β, TNF-α, and IL-6; MaR-1 suppresses glial activation, reducing inflammation and pain sensitivity.^3^ In radicular pain models, MaR-1 inhibits NLRP3 inflammasome activation and pyroptosis, preventing pain-related cellular processes.^4^ Maresin-1 treatment restores synaptic proteins such as PSD95 and synapsin II disrupted after nerve injury, enhancing synaptic transmission. It also inhibits TRPV1 ion channels and suppresses Calcitonin gene-related peptide (CGRP) release from nociceptors, key steps in pain perception.^5^ Additionally, MaR-1 inhibits NF-κB signaling, reducing transcription of pro-inflammatory genes and inflammation-induced pain.^4^ Intrathecal MaR-1 administration in rat nerve injury models significantly alleviates mechanical and thermal hyperalgesia.^3^

In rheumatoid arthritis (RA), pain is predominantly inflammatory; however, many patients also experience neuropathic pain characterized by burning, electric shocks, or tingling sensations. Neuropathic pain may result from chronic synovitis affecting peripheral nerves mechanically or via inflammatory compression, and prolonged inflammation can induce central sensitization leading to persistent pain. Cytokines such as TNF-α and IL-1β further sensitize nerve endings and amplify neuropathic pain.^6^^-^^8^

Fibromyalgia (FM) is a chronic pain syndrome presenting with diffuse musculoskeletal pain, fatigue, sleep disturbance, and cognitive dysfunction. Growing evidence suggests a significant neuropathic component in FM pain, with many patients reporting burning, tingling, or numbness. Approximately 40%-50% of FM patients show small fiber neuropathy affecting unmyelinated fibers responsible for transmitting pain, temperature, and touch, contributing to neuropathic-like symptoms. Central sensitization also plays a role, rendering the central nervous system hypersensitive and causing allodynia and hyperalgesia, while elevated inflammatory markers may contribute to neuropathic pain.^9^

Although RA is a prototypical inflammatory arthritis and FM is traditionally considered a non-inflammatory pain syndrome, both conditions share overlapping pain phenotypes.^8^^,^^10^ Maresin-1, a lipid mediator with anti-inflammatory and neuroprotective properties, has emerged as a promising candidate for resolving both inflammatory and neuropathic pain.^4^ Its ability to suppress glial activation, modulate inflammatory pathways, and reduce neuronal hypersensitivity underlies its long-lasting analgesic effects, making MaR-1 a potential next-generation strategy for chronic pain management.

This study aimed to compare serum MaR-1 levels, thought to play a role in inflammatory processes, between RA and FM patients—2 conditions frequently associated with chronic pain—to clarify its potential as a biomarker of neuroinflammation. It was hypothesized that MaR-1 levels would be lower in both patient groups compared with healthy controls and would inversely correlate with pain severity and disease activity measures.

## Materials and Methods

### Patient Population

The study enrolled 30 patients diagnosed with RA and 30 with FM, all under regular follow-up for at least 1 year. The participants were recruited between February 2025 and August 2025 at the Department of Physical Medicine and Rehabilitation. All RA patients met the 2010 ACR/EULAR classification criteria for RA, and FM patients fulfilled the 2010 ACR criteria for FM.^11^^,^^12^ A total of 29 age- and sex-matched healthy individuals served as controls. All RA patients underwent clinical evaluation, and individuals with a known diagnosis of fibromyalgia were excluded from the RA cohort in order to minimize potential confounding effects on pain perception and symptom severity. An a priori power analysis was conducted using G*Power 3.1.9.4 (Heinrich-Heine-Universität; Düsseldorf, Germany) with *α* = 0.05, power = 0.80, and an expected medium effect size (f = 0.25), indicating that a minimum of 28 participants per group (RA, FM, and healthy controls) was required. The expected effect size was chosen based on moderate between-group differences reported in previous multi-group comparisons involving RA, FM, and healthy control cohorts. Exclusion criteria included any change in Disease-Modifying Antirheumatic Drugs (DMARD) or biologic therapy within the previous 3 months (except dosage adjustments), comorbid autoimmune disorders other than RA, active or chronic infections, malignancies, or significant pulmonary, hepatic, renal, or endocrine disease. Participants were excluded if they were current smokers, had hypertension (defined as blood pressure ≥140/90 mmHg or use of antihypertensive medication), had hypercholesterolemia, or were outside the 20-70-year age range. For RA patients, DMARD type, biologic agent, dosage, and treatment duration were documented. For FM patients, the use of analgesics or neuromodulators (e.g., pregabalin, duloxetine) was recorded to account for potential confounders. All participants provided written informed consent before inclusion. The study complied with the Declaration of Helsinki (2008) and was approved by the Elazığ Fethi Sekin City Hospital Non-Interventional Clinical Research Ethics Committee (Approval No: 776393C3-0273-4FE7-8A8C-5B70E98AB549; Date: February 12, 2025).

Demographic characteristics (age, sex, and body mass index (BMI)) were recorded for all participants at enrollment; BMI was calculated as weight (kg) divided by height squared (m²). In the RA group, disease duration was defined as years since diagnosis and obtained from medical records. In the FM group, disease duration and the duration of widespread pain were recorded in years; the duration of widespread pain was defined as the time since the onset of widespread pain symptoms based on participant self-report.

### Clinical Assessment

Pain and fatigue severity were assessed using a visual analog scale (VAS).^13^ In the FM group, disease impact was assessed using the Fibromyalgia Impact Questionnaire (FIQ); higher scores indicate greater impact/severity.^14^,^15^ Disease activity in RA was measured using the Disease Activity Score 28 (DAS28),^16^ with DAS28 ≤ 3.2 classified as low disease activity and values > 3.2 as moderate/high activity.^17^ In the RA group, neuropathic pain features were screened using the PainDETECT questionnaire (total score range: 0-38). For categorical analyses, participants with a PainDETECT total score ≥19 were classified as having possible neuropathic pain, whereas scores <19 were considered not suggestive of neuropathic pain.^18^ Emotional status was assessed using the Beck Anxiety Inventory (BAI) and the Beck Depression Inventory (BDI) in the RA and FM groups; higher scores indicate greater symptom severity.^19^^-^^22^

### Laboratory Evaluations

Standard laboratory investigations included erythrocyte sedimentation rate (ESR: mm/h), C-reactive protein (CRP; mg/L), rheumatoid factor (RF; IU/mL), anti–cyclic citrullinated peptide antibody (anti-CCP; U/mL). Rheumatoid factor and CRP levels were measured by nephelometry.

### Serum Maresin-1 Assay

Fasting venous blood samples were collected in the morning into serum separator tubes, allowed to clot at room temperature for 30 minutes, and centrifuged at 1200 × g for 10 minutes. The supernatant serum was transferred to Eppendorf tubes and stored at −80°C until analysis. Serum MaR-1 concentrations were determined using a commercial ELISA kit (Bioassay Technology Laboratory, Shanghai, China). The kit detection range was 5-500 ng/L, with intra-assay CV < 10% and inter-assay CV < 12%. Assays were performed according to the manufacturer’s protocol, and absorbance was read at 450 nm, and results were expressed in ng/L.

### Statistical Analysis

Data normality was tested with the Shapiro–Wilk test. For 2-group comparisons, the independent samples *t*-test was applied for normally distributed variables, and the Mann–Whitney *U-*test for non-normally distributed data. For 3-group comparisons, 1-way ANOVA or the Kruskal–Wallis *H-*test was used, depending on distribution. Homogeneity of variances was evaluated using Levene’s test; when violated, Welch’s ANOVA was used. Post hoc analyses employed Tamhane T2 for parametric data and Bonferroni-corrected Dunn’s test for nonparametric data. Fisher’s exact test was used for categorical variables when appropriate.^^23^^ Correlations between non-normally distributed MaR-1 levels and continuous variables within RA and FM groups were assessed using Spearman’s rho. The discriminative ability of MaR-1 levels in this cohort was explored using receiver operating characteristic (ROC) analysis.^24^ Optimal cut-off points were determined based on the best balance between sensitivity and specificity. Descriptive statistics are presented as mean ± standard deviation or median (min-max) for continuous variables and frequency (n) and percentage (%) for categorical variables. Statistical significance was set at *P* < .05. Analyses were performed using IBM SPSS Statistics for Windows, Version 26.0 (IBM SPSS Corp.; Armonk, NY, USA)

## Results

There were no significant differences among the groups regarding age, sex, BMI, or disease duration (all *P* > .05). Among RA patients, 46.6% (n = 14) had high disease activity, and 43.3% (n = 13) were identified as having a possible neuropathic pain component. Visual analog scale pain and fatigue scores, as well as the duration of widespread pain (years), were markedly higher in the FM group compared with both RA patients and healthy controls (all *P* < .05). Serum MaR-1 levels were significantly higher in the control group than in either patient group (*P* < .001). The demographic and clinical characteristics of the study population are summarized in Table 1.

When RA patients were stratified by disease activity, those with high disease activity had significantly elevated ESR, CRP, VAS pain, fatigue, and PainDETECT scores (all *P* <.05), whereas serum MaR-1 levels were substantially lower in this subgroup (*P* < .001). The demographic and clinical characteristics of RA patients according to disease activity status are presented in Table 2.

Analysis of RA patients based on the presence of possible neuropathic pain revealed significantly higher CRP levels (*P* = .018), VAS fatigue scores (*P* = .008), and DAS28 scores (*P* = .013) in those with possible neuropathic pain. Serum MaR-1 levels were significantly reduced in this group (*P* = .008). Table 3 provides the intergroup comparisons according to neuropathic pain status.

Correlation analysis demonstrated a significant negative association between serum MaR-1 levels and VAS pain (r = −0.759, *P* < .001), DAS28 (r = −0.683, *P* < .001) in RA patients. In FM patients, MaR-1 levels showed a significant negative correlation with VAS pain (r = −0.614, *P* < .001). These results are summarized in Table 4.

**Figure 1:**
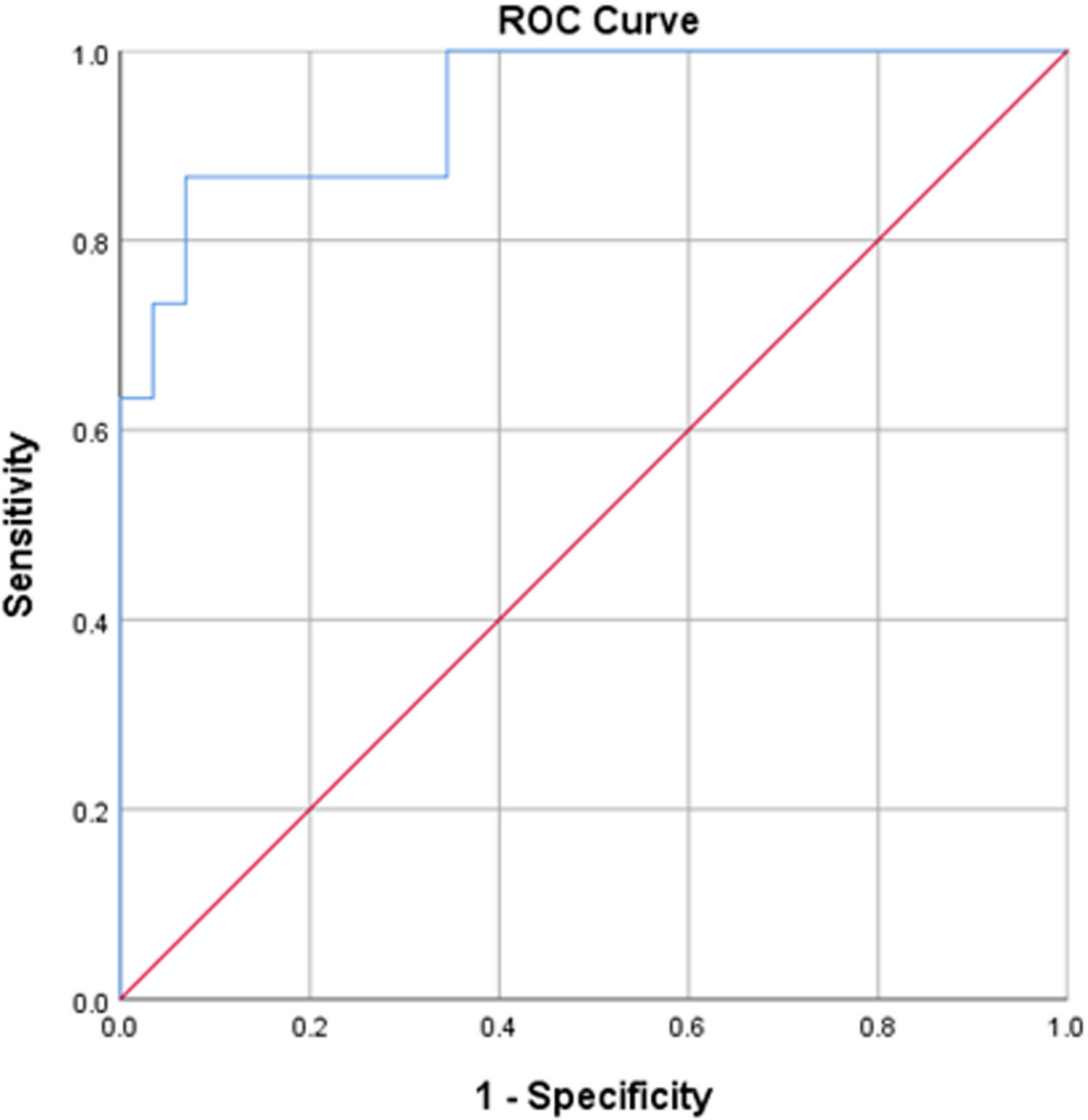
ROC Curve for MaR-1 Levels in Rheumatoid Arthritis.

**Figure 2:**
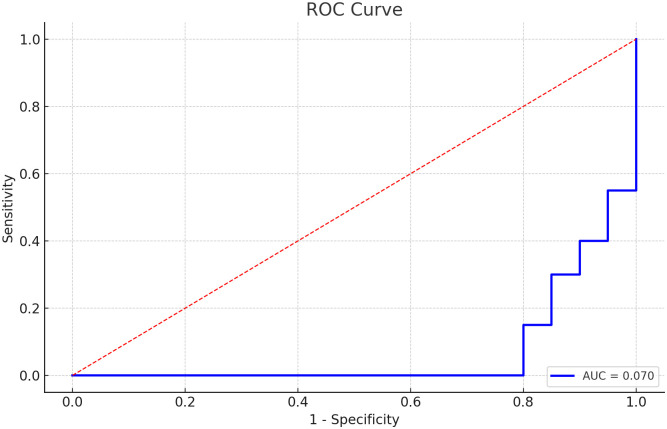
ROC Curve for MaR-1 Levels in Fibromyalgia.

To assess the discriminative ability of serum MaR-1 levels in this cohort, exploratory ROC analyses were conducted for both patient groups. For RA patients, the cut-off value was ≤ 1.07, yielding an AUC (95% CI) of 0.941 (0.887-0.996), sensitivity of 86.7%, and specificity of 93.1%. For FM patients, the cut-off value was ≤ 1.19, yielding an AUC (95% CI) of 0.933 (0.861-1.000), sensitivity of 90.0%, and specificity of 93.1%. The ROC curves and related findings are displayed in Figures 1 and 2 and summarized in Table 5. These cut-off values are exploratory and are not intended for direct clinical diagnostic use without external validation.

## Discussion

In this cross-sectional study, serum MaR-1 levels were lower in patients with RA and FM than in healthy controls and were associated with indices of disease activity and symptom burden. In RA, MaR-1 levels tended to be lower among patients with higher disease activity and in those with possible neuropathic pain features. In correlation analyses, MaR-1 showed inverse associations with DAS28, VAS pain in RA, and with VAS pain in FM.

Rheumatoid arthritis is typically defined by synovitis and pain primarily driven by inflammation. Nevertheless, inflammation and structural joint damage are not the only sources of pain in RA. Earlier investigations have shown that synovial inflammation promotes prostaglandin and bradykinin production, which subsequently activate unmyelinated sensory C-fibers within the synovium.^25^ While inflammation is a key contributor to RA-related pain, several reports describe persistent pain even after inflammation is adequately controlled with anti-inflammatory therapies, suggesting the presence of additional pain mechanisms.^2^ Experimental evidence has demonstrated that MaR-1 can suppress neuroinflammation and glial activation in neuropathic pain models, thereby reducing central sensitization and behavioral pain responses.^27^-^29^ Only a limited number of studies have examined the relationship between MaR-1 and rheumatoid arthritis. Available data suggest that MaR-1 levels are generally higher in patients with inactive RA and inversely related to disease activity.^30^ Jin et al found markedly higher serum MaR-1 levels in inactive RA and significantly reduced levels in active disease. In their analysis, FoxP3 expression (a regulatory T-cell marker) was greatest in inactive RA and lowest in active RA, whereas RORc (a Th17-related transcription factor) showed the opposite trend. They also reported an inverse correlation between the FoxP3/RORc ratio and DAS28 scores. These observations suggest that MaR-1 may influence disease activity by modulating the Treg/Th17 balance in RA. Consistent with these findings, the study also demonstrated a negative correlation between serum MaR-1 levels and DAS28 scores.^30^ As an exploratory approach to support the study objective, MaR-1 was examined across subgroups defined by RA disease activity and PainDETECT status to better contextualize its relationship with inflammatory burden and symptom profile. Stratification of RA patients by disease activity suggested a clear clinical pattern: those with high disease activity had higher ESR and CRP levels, accompanied by greater pain intensity, fatigue, and higher PainDETECT scores, whereas serum MaR-1 concentrations tended to be lower. These findings are consistent with the possibility that reduced availability of pro-resolving mediators may co-occur with periods of heightened inflammatory activity and increased symptom burden. Similarly, RA patients with possible neuropathic pain features showed higher CRP levels, higher DAS28 scores, and greater fatigue, together with lower MaR-1 concentrations, indicating that neuropathic-like symptoms may be observed alongside higher inflammatory activity in this sample. However, these subgroup analyses are exploratory and do not allow causal inference; nonetheless, they provide hypothesis-generating evidence that helps place MaR-1 in a clinically relevant context as a candidate biomarker related to both disease activity and pain phenotype. Beyond its anti-inflammatory activity, MaR-1 has been implicated in pain modulation. Serhan et al reported that capsaicin and vincristine administration to the dorsal root ganglion induced neuropathic, TRPV1-mediated pain, which was reversed by MaR-1 treatment.^31^ Additional experimental work has shown that MaR-1 alleviates allodynia and thermal hyperalgesia by decreasing neuronal hypersensitivity.^3^ Maresin-1 has also been reported to dampen dorsal root ganglion neuron activation and inhibit CGRP release, effects that likely contribute to its partial analgesic and anti-inflammatory actions in chronic pain models.^5^ Given its role in actively resolving inflammation, MaR-1 may represent a potential therapeutic target in RA and a possible adjunct to current treatment strategies.

Although studies examining serum MaR-1 levels in FM patients remain scarce, the available literature offers several valuable insights. Increasing evidence indicates that neuroinflammation plays a pivotal role in both the onset and persistence of fibromyalgia symptoms, highlighting MaR-1 as a molecule of potential relevance. For example, in an experimental autoimmune encephalomyelitis model, administration of MaR-1 reduced neuroinflammation and improved neurological outcomes.^32^ Another investigation reported that MaR-1 enhanced IL-10 production and limited the infiltration of inflammatory cells into the central nervous system. MaR-1 was also shown to restore metabolic balance in CD4+ T cells, macrophages, and microglia, while improving mitochondrial function in oligodendrocytes—neuroprotective actions achieved partly through the suppression of glycolysis.^33^ Furthermore, MaR-1 has been demonstrated to reduce mechanical hypersensitivity and inhibit microglial and astrocytic activation in spinal cord injury–induced neuropathic pain models.^34^ Additionally, intracerebroventricular MaR-1 administration was found to attenuate lipopolysaccharide-induced depression-like behavior.^28^ In FM, the observed inverse associations between serum MaR-1 levels and VAS pain are compatible with MaR-1 as a preliminary candidate marker related to symptom burden; however, these findings require external validation before any clinical or therapeutic inferences can be made.

Despite the exclusion of patients with a diagnosis of fibromyalgia from the RA group, relatively high Beck depression and anxiety scores and a notable prevalence of neuropathic pain features were observed in the RA cohort. This finding may reflect the multifactorial nature of pain in rheumatoid arthritis. Beyond peripheral inflammatory nociception, increasing evidence suggests that central sensitization mechanisms may also contribute to pain perception in RA, leading to neuropathic pain–like symptoms even in the absence of concomitant fibromyalgia. Indeed, previous studies have reported neuropathic pain features in approximately 20%-40% of RA patients, indicating that non-nociceptive pain mechanisms may play a substantial role in this population. In addition, chronic pain, functional impairment, and persistent systemic inflammation are known to be associated with increased psychological distress, including depression and anxiety, in patients with RA. Therefore, the elevated PainDETECT scores and psychological symptom burden observed in the study may reflect alterations in central pain processing and the psychosocial burden of chronic inflammatory disease, rather than the presence of comorbid fibromyalgia. Nevertheless, the possibility of subclinical central sensitization in some RA patients cannot be completely excluded.

The present findings suggest that MaR-1 may be informative when interpreted alongside routine measures of inflammatory activity and symptom burden. In RA, combining disease activity indices (e.g., DAS28, ESR/CRP) with pain phenotyping (e.g., PainDETECT) and patient-reported symptom severity (pain and fatigue) may help contextualize persistent pain complaints and support a more individualized, multidisciplinary management approach. In rehabilitation settings, identifying patients with higher fatigue and possible neuropathic pain features may be relevant for tailoring exercise dosing, education, and self-management strategies, and considering referral to multidisciplinary pain services when appropriate. In FM, the association between lower MaR-1 levels and higher symptom burden is consistent with the possibility of altered pro-resolving pathways in chronic pain phenotypes. Importantly, these implications are exploratory and hypothesis-generating; MaR-1 is not proposed as a stand-alone clinical test, and further longitudinal and interventional studies are needed before routine clinical implementation.

One of the strengths of this study is that it assessed MaR-1 levels in 2 related yet distinct chronic pain conditions. Nevertheless, several limitations should be considered. Given that anxiety and depressive symptoms can affect pain perception, emotional status in the patient groups was evaluated using the BAI and the BDI. In the sample, BAI and BDI scores were similar between the RA and FM groups, indicating that the observed between-group differences in MaR-1 are unlikely to be attributable solely to differences in emotional distress. PainDETECT was not administered in the fibromyalgia group, as fibromyalgia is currently considered a nociplastic pain condition, whereas the PainDETECT questionnaire was originally developed to screen for neuropathic pain related to lesions or diseases of the somatosensory system. Nevertheless, previous studies have shown that neuropathic pain–like symptoms may also be present in patients with fibromyalgia. Therefore, the absence of PainDETECT assessment in the FM group represents a limitation of the present study, and future studies may benefit from evaluating neuropathic pain features across both conditions. The relatively small sample size and the single-center, cross-sectional design limit the generalizability of the findings and do not allow for causal inference. Given the small, single-center cross-sectional sample and the absence of an independent validation cohort, the high AUC values may be influenced by overfitting and spectrum effects (spectrum bias). Therefore, the ROC analyses should be interpreted as hypothesis-generating rather than evidence of clinical diagnostic validity. In addition, medication use represents a major potential confounder; although DMARD/biologic exposure in RA and analgesic/neuromodulatory treatments in FM were documented, these variables were not included as covariates in the analyses, and residual confounding cannot be excluded. Potential confounders—such as lifestyle factors (e.g., diet, smoking history), and subtle variations in comorbidity profiles—were not fully controlled. Furthermore, only serum MaR-1 concentrations were evaluated, and neither tissue-level measurements nor longitudinal changes were examined. Future larger studies should incorporate treatment-related covariates to clarify the independent association of MaR-1 with disease activity and pain phenotype.

This study found that serum MaR-1 levels were lower in patients with RA and FM than in healthy controls and were associated with greater pain burden and indices of disease activity. In RA, lower MaR-1 levels were observed in patients with higher disease activity and in those with possible neuropathic pain features, whereas in FM reduced MaR-1 was seen in individuals with higher symptom burden. Overall, these findings are consistent with the possibility that alterations in pro-resolving lipid mediator pathways may be linked to inflammatory activity and chronic pain phenotypes. Maresin-1 may therefore represent a candidate biomarker for patient stratification and disease monitoring; however, any potential biomarker utility should be considered preliminary pending independent external validation. Longitudinal, multicenter studies are warranted to clarify temporal changes in MaR-1 in relation to disease fluctuations and treatment responses.

### Data Availability Statement:

The data that support the findings of this study are available on request from the corresponding author.

### Artificial Intelligence Usage Statement:

The authors declare that no generative artificial intelligence (AI) or large language model (LLM) tools were used in the preparation, writing, or editing of this manuscript.

### Ethics Committee Approval:

Ethical committee approval was received from Elazığ Fethi Sekin City Hospital Non-Interventional Clinical Research Ethics Committee (Approval No: 776393C3-0273-4FE7-8A8C-5B70E98AB549; Date: February 12,2025).

### Informed Consent:

Written informed consent was obtained from the patients who agreed to take part in the study.

### Peer-review:

Externally peer-reviewed.

### Acknowledgment:

The authors would like to thank Prof. Dr. Özkan Timurkan for his assistance at the laboratory analyses stage.

### Author Contributions:

Concept – T.T., Ş.D.; Design – T.T., N.P.T.; Supervision – T.T., N.P.T., M.Ş.E.; Materials – T.T., N.P.T., M.Ş.E., Ş.D.; Data Collection and/or Processing – T.T., N.P.T.; Analysis and/or Interpretation – Ş.D., T.T.; Literature Search – T.T., N.P.T., M.Ş.E., Ş.D.; Writing – T.T., N.P.T., M.Ş.E.; Critical Review – T.T., M.Ş.E.

### Declaration of Interests:

The authors have no conflicts of interest to declare.

### Funding:

The authors declare that this study received no financial support.

## Figures and Tables

**Table 1.** Evaluation of Demographic and Clinical Characteristics by Group

**Variable**	**Control (n = 29)**	**Rheumatoid Arthritis (n = 30)**	**Fibromyalgia (n = 30)**	***P***
Age (years)	44.83 ± 17.51^a^^b^	43.97 ± 12.87^a^	42.57 ± 10.34^b^	.256
Sex, n (%)	​	​	​	1.000^f^
Female	23 (79.3%)	24 (80%)	24 (80%)	​
Male	6 (20.7%)	6 (20%)	6 (20%)	​
BMI (kg/m²)	26.5 (18.9-34.6)	26.8 (19.8-32.8)	26.9 (18.2-35.3)	.544^m^
Disease duration (years)	–	7.67 ± 5.72	6.56 ± 4.32	.254
Disease activity, n (%)	–	​	–	​
Low	–	16 (53.3%)	–	​
High	–	14 (46.7%)	–	​
Neuropathic pain, n (%)	–	​	​	​
Possible	–	13 (43.3%)	14 (46.7%)	​
Not possible	–	17 (56.7%)	16 (53.3%)	​
VAS pain	–	4 (2-8)	6 (2-8)	.031^m^
VAS fatigue	–	4.6 ± 2.01	5.8 ± 1.58	.027
Duration of widespread pain (years)	–	1.8 ± 2.42	3.9 ± 2.31	.004
FIQ	–	–	52.7 ± 15.45	–
Beck Anxiety Inventory Score	–	26.3 ± 3.87	28.5 ± 10.15	.214
Beck Depression Inventory Score	–	31.6 ± 9.28	32.0 ± 10.28	.415
DAS28	–	3.08 ± 1.48	–	–
PainDETECT	–	13.7 ± 10.17	–	–
ESR (mm/h)	17 (4-65)^a^	23 (2-65)^a^	8 (4-20)^b^	<.001^h^
CRP (mg/L)	3.13 (3.13-7.15)^a^	8.75 (3-42)^b^	2.63 (1.2-11.6)^a^	<.001^h^
Anti-CCP (U/mL)	0.5 (0.5-0.5)	18.5 (0.5-298)	–	<.001^m^
RF (IU/mL)	9.38 (9.38-9.38)	26.5 (8-232)	–	.158^m^
MaR-1 (ng/L)	6.57 (3.12-8.57)^a^	2.54 (0.84-6.8)^b^	1.6 (0.26-2.6)^b^	<.001^h^

Values are presented as: mean ± standard deviation; median (min–max); or number (%), as appropriate. Statistical tests used: ^w^Welch’s test, ^h^Kruskal-Wallis H test, ^m^Mann–Whitney U test, ^f^Fisher’s exact test Superscript letters (a, b): Groups sharing the same letter are not significantly different from each other (based on post-hoc Tamhane T2 or Bonferroni-corrected Dunn’s test). Test stat.: Indicates test statistic used for the *P*-value.

Anti CCP, anti–cyclic citrullinated peptide antibodies; CRP, C-reactive protein; DAS28, Disease Activity Score 28; ESR, erythrocyte sedimentation rate; FIQ, Fibromyalgia Impact Questionnaire; MaR-1, maresin-1; RF, rheumatoid factor; VAS, Visual Analog Scale.

**Table 2.** Demographic and Clinical Characteristics of RA Patients According to Disease Activity

**Variable**	**Low Disease Activity (n = 16)**	**High Disease Activity (n = 14)**	***P***
Age (years)	51.06 ± 13.6	57.29 ± 11.57	.191^t^
Sex, n (%)	​	​	1.000^f^
Female	13 (81.3%)	11 (78.6%)	​
Male	3 (18.8%)	3 (21.4%)	​
BMI (kg/m²)	27.02 ± 4.03	25.72 ± 3.34	.350^t^
Disease duration (years)	5.5 (1-24)	7.5 (1-22)	.295^m^
ESR (mm/h)	20 (3-48)	39 (2-65)	.037^m^
CRP (mg/L)	3.3 (3-17)	15.1 (3-42)	.001^m^
Anti-CCP (U/mL)	18.5 (0.5-200)	19 (0.5-298)	.877^m^
RF (IU/mL)	12.5 (9-232)	43.5 (8-175)	.639^m^
VAS Pain	3 (2-5)	7 (3-8)	<.001^m^
VAS Fatigue	4 (3-5)	6 (4-9)	<.001^m^
MaR-1 (ng/L)	4.39 (2.55-6.8)	1.02 (0.84-2.56)	<.001^m^
DAS28	2.37 (0.8-3.06)	4.24 (0.53-6.17)	<.001^m^
PainDETECT	4 (0-14)	20 (4-35)	.007^m^

Values are presented as: mean ± standard deviation, median (min–max), or n (%), as appropriate. Statistical tests used: ^t^Independent Samples t-test, ^m^Mann–Whitney *U-*test, ^f^Fisher’s exact test.

Anti CCP, anti–cyclic citrullinated peptide antibodies; BMI, body mass index; CRP, C-reactive protein; DAS28, Disease Activity Score 28; ESR, erythrocyte sedimentation rate; MaR-1, maresin-1; RF, rheumatoid factor; VAS, Visual Analog Scale.

**Table 3.** Clinical and Demographic Characteristics of RA Patients Based on Neuropathic Pain Status

**Variable**	**Possible Neuropathic Pain (n = 13) PainDETECT ≥19**	**Not Possible (n = 17) PainDETECT <19**	***P***
Age (years)	57.77 ± 13.98	51.06 ± 11.52	.161^t^
Sex, n (%)	​	​	.196^f^
Female	12 (92.3%)	12 (70.6%)	​
Male	1 (7.7%)	5 (29.4%)	​
BMI (kg/m²)	25.58 ± 4.4	27.05 ± 3.1	.319^t^
Disease duration (years)	8 (2-22)	7 (1-24)	.342^m^
ESR (mm/h)	29.23 ± 19.46	28.53 ± 20.98	.926^t^
CRP (mg/L)	15 (3-42)	3.3 (3-31)	.018^m^
Anti-CCP (U/mL)	200 (0.5-298)	18 (0.5-200)	.386^m^
RF (IU/mL)	45 (8-232)	9 (9-206)	.356^m^
VAS pain	6 (2-8)	3 (2-8)	.055^m^
VAS fatigue	5 (3-8)	4 (3-9)	.008^m^
MaR-1 (ng/L)	1.28 (0.84-3.56)	6.59 (1.4-6.8)	.008^m^
DAS28	3.83 ± 1.54	2.51 ± 1.17	.013^t^
PainDETECT	21 (19-35)	6 (0-18)	<.001^m^

Values are presented as: mean ± standard deviation; median (min–max); or n (%), as appropriate. Statistical tests used, ^t^Independent Samples t-test, ^m^Mann–Whitney U test, ^f^Fisher’s exact test.

Anti CCP, anti–cyclic citrullinated peptide antibodies; BMI, body mass index; CRP, C-reactive protein; DAS28, Disease Activity Score 28; ESR, erythrocyte sedimentation rate; MaR-1, maresin-1; RF, rheumatoid factor; VAS, visual analog scale.

**Table 4.** Correlation Between Serum MaR-1 Levels and Quantitative Variables in RA and FM Patients

**Variable**	**RA (r)**	**RA (p)**	**FM (r)**	**FM (p)**
Age	0.218	0.246	0.367	0.046
BMI (kg/m²)	−0.168	0.375	0.109	0.566
VAS Pain	−0.759	<0.001	-0.614	<0.001
DAS28	−0.683	<0.001	–	–
PainDETECT	−0.035	>0.05	–	–
ESR (mm/h)	0.327	0.078	0.259	0.167
CRP (mg/L)	0.558	0.001	0.016	0.932
Anti-CCP (U/mL)	0.073	0.701	–	–
RF (IU/mL)	0.193	0.308	–	–
FIQ	–	–	-0.039	>0.05

r: Spearman’s rho correlation coefficient. *P*: *P*-value indicating the statistical significance of the correlation.

Anti CCP, anti–cyclic citrullinated peptide antibodies; BMI, body mass index; CRP, C-reactive protein; DAS28, Disease Activity Score 28; ESR, erythrocyte sedimentation rate; FIQ, Fibromyalgia Impact Questionnaire; MaR-1, maresin-1; RF, rheumatoid factor; VAS, Visual Analog Scale.

**Table 5.** Exploratory ROC Analysis Results for MaR-1 Levels in RA and FM Patients

​	**AUC (95% CI)**	***P***	**Cut-off**	**Sensitivity (%)**	**Specificity (%)**
MaR-1 in RA	0.941 (0.887-0.996)	<.001	≤ 1.07	86.7	93.1
MaR-1 in FM	0.933 (0.861-1.000)	<.001	≤ 1.19	90.0	93.1

FM, fibromyalgia; MaR-1, maresin-1; RA, rheumatoid arthritis.
